# Predictive Factors for Ultrasonographic Grading of Nonalcoholic Fatty Liver Disease

**DOI:** 10.5812/hepatmon.6860

**Published:** 2012-11-04

**Authors:** Mohammad Ebrahim Ghamar-Chehreh, Hossein Khedmat, Mohsen Amini, Saeed Taheri

**Affiliations:** 1 Baqiyatallah Research Center for Gastroenterology and Liver Disease, Baqiyatallah University of Medical Sciences, Tehran, IR Iran; 2 Medical Research Group, Baqiyatallah University of Medical Sciences, Tehran, IR Iran

**Keywords:** Ultrasonography, Non-Alcoholic Fatty Liver Disease

## Abstract

**Background:**

There are several studies in the literature investigating factors which can induce non-alcoholic fatty liver disease (NAFLD) in different populations. However, the existing literature lacks powerful studies addressing the factors which may predict the severity of NAFLD.

**Objectives:**

In the current study, we aimed to evaluate factors independently associated with liver echogenicity in an Iranian NAFLD patient population.

**Patients and Methods:**

A total of 393 patients attending as outpatients at the Hepatology Clinic of Baqiyatallah University of Medical Sciences were entered into this analysis. Univariate and multivariable linear regression models were performed to evaluate the effects of the study variables on the NAFLD grade, defined by ultrasound hepatic echogenicity.

**Results:**

Univariate linear analyses revealed a significant relationship between; the ultrasonographic grading of NAFLD and body weight (P < 0.001), abdominal girth (P = 0.007), pelvic girth (P = 0.032), fasting blood glucose (FBS) (P = 0.005), serum insulin (P = 0.035), hemoglobin A1c (HbA1c) (P = 0.012), triglycerides (P = 0.049), aspartate aminotransferase (AST) (P = 0.015), alanin aminotransferase (ALT) (P = 0.026), and homeostasis model assessment (HOMA) (P = 0.002). Multivariable linear regression models left only; HbA1C (P = 0.011, β = 0.133), body weight (P = 0.001; β = 0.176) and serum triglyceride (P = 0.034; β = 0.112) as factors independently associated with liver echogenicity.

**Conclusions:**

Diabetic patients can reduce liver damage of NAFLD with control of their HbA1C through the lower ranges. Hypertriglyceridemia and body weight are the other implicated factors, which worsen hepatic echogenicity in the NAFLD patient population. We recommend future prospective studies and clinical trials to confirm our findings.

## 1. Background

Nonalcoholic fatty liver disease (NAFLD) is the terminology used for a wide spectrum of disorders ranging from; non-evaluative simple steatosis to progressive nonalcoholic steatohepatitis (NASH) and cirrhosis (1). The pathogenesis of NAFLD is multifactorial, and has not been fully elucidated (2). The main mechanism through which NAFLD is induced is insulin resistance; a metabolic state in which normal insulin serum concentrations induce an inadequate response, or to attain normal blood glucose levels, we need higher than normal insulin concentrations. This state is a predictor of diabetes mellitus, which is a disease with a wide range of morbidity and mortality. Epidemiological studies have highlighted disparities in the characteristics and features of NAFLD disease regarding specific ethnic populations and geographical regions. Explanations for this observation are; inconsistencies in the lifestyles and eating habits, in different regional areas (3). Putting these factors together, we felt that it would be worthwhile if a study was conducted to address the elements associated with higher liver involvement of NAFLD in our patients.

## 2.Objectives

This study was conducted in order to evaluate potential independent effects of some of the most likely factors responsible for the promotion of NAFLD grade development, in our relatively large Iranian NAFLD patient population.

## 3. Patients and Methods

This was a cross-sectional study, conducted in the outpatients Hepatology Clinic of Baqiyatallah University of Medical Sciences, Tehran, Iran. During a period of two years, from April 2009 to March 2011, a total of 393 patients who attended our clinic with a diagnosis of NAFLD, were consecutively entered into the analysis. Exclusion criteria were; an active HBV infection, having HCV positive serology, and corticosteroid therapy. Metabolic syndrome was diagnosed according to the third report of the Expert Panel on Detection, Evaluation, and Treatment of High Blood Cholesterol in Adults (ATP III) (4). Homeostatic model assessment (HOMA) was calculated using the following formula; insulin (mIU/L)* glucose (mmol/L)/22.5. For the continuous data, a Student’s t-test was used. A Chi-square test was used for the categorical analysis, and P < 0.05 was considered significant. Univariate and multivariable linear regression models were performed to evaluate the effects of the study variables on NAFLD grades, defined by ultrasound hepatic echogenicity. Statistical analyses were performed using SPSS-17.0 (SPSS Corp.; IL; Chicago; USA) for Windows.

## 4. Results

A total of 393 patients were entered into the analysis. There were 229 (58.3%) males and 164 (41.7%) females. The mean ± SD age of the study population was 45.5 ± 12.8 years, and 44 (11.2%) of the participants were diabetic. Table 1 summarizes the associations of ultrasonographic-based NAFLD grading. There were significant associations between liver echogenicity and body weight (r = 0.201, P < 0.001), abdominal girth (r = 0.136, P = 0.007), pelvic girth (r = 0.109, P = 0.032), FBS (r = 0.119, P = 0.035), serum insulin (r = 0.199, P < 0.001), HbA1C (r = 0.134, P = 0.012), triglycerides (r = 0.099, P = 0.049), AST (r = 0.123, P = 0.015), ALT (r = 0.113, P = 0.026), and HOMA (r = 0.169, P = 0.002). Linear regression analyses were performed to evaluate the magnitude of the correlations between the study variables and ultrasonographic grading of the NAFLD. Univariate analyses (Table 2) revealed a significant direct relationship between ultrasonographic grading of the NAFLD and; body weight (r = 0.011), abdominal girth (r = 0.009), pelvic girth (r = 0.008), FBS (r = 0.003), serum insulin (r = 0.044), HbA1C (r = 0.025), triglycerides (r = 0.001), AST (r = 0.005), ALT (r = 0.003), and HOMA (r = 0.266). On the other hand, no relationship was found for the blood pressure indices, and serum cholesterol levels (P > 0.2 for both). Multivariable linear regression models were used to evaluate the independence of the results. In the first model, we entered study variables and HbA1c (P = 0.011; r = 0.03), triglycerides (P = 0.034; r = 0.001) and weight (P = 0.001; r = 0.12), saved their significance and represented the independent relationship with NAFLD grading. Then to evaluate whether serum insulin or FBS can affect NAFLD severity, HOMA was replace by these two variables in a new multivariable linear regression model, however, none of these represented an independent significant effect.

**Table 1:** Demographic and Laboratory Measures of Study Participants With Regard to Their NAFLD Grade

	NAFLD Ultrasonographic Grading				P value
	Normal	I	II	III	
**Gender**	8 (72.7)	97 (51.3)	84 (61.3)	40 (71.4)	0.027
**Diabetes mellitus, No. (%)**	1(9.1)	10 (5.3)	23 (16.8)	10 (17.9)	0.004
**Coronary artery disease, No. (%)**	1 (9.1)	13 (6.9)	13 (9.5)	0	0.128
**Hypertension, No. (%)**	0	46 (24.5)	28 (20.6)	11 (19.6)	0.248
**Insulin resistance, No. (%)**	5 (62.5)	83 (51.9)	70 (63.6)	35 (75.5)	0.02
**Abdominal obesity, No. (%)**	5 (45.5)	127 (68.3)	96 (70.1)	39 (73.6)	0.322
**Metabolic syndrome, No. (%)**	3 (27.3)	63 (33.3)	57 (41.6)	29 (51.8)	0.059
**BMI, Kg/m^2^, Mean ± SD**	28 ± 5.1	29.4 ± 4.6	30 ± 4.4	29.8 ± 4.5	0.08
**HOMA, Mean ± SD**	2.9 ± 1.8	2.9 ± 2.3	3.6 ± 2.5	4.5 ± 4	0.005
**Age, y, Mean ± SD**	43.9 ± 15.2	45.8 ± 13.5	45.9 ± 12.7	43.7 ± 10.1	0.666
**Systolic blood pressure, mm/Hg, Mean ± SD**	117.3 ± 15.6	123.7 ± 18.1	125.2 ± 16.4	124.2 ± 12	0.469
**Diastolic blood pressure, mm/Hg, Mean ± SD**	76.4 ± 11.2	80.8 ± 8.4	80.7 ± 8	81.7 ± 7.5	0.278
**Weight, Kg, Mean ± SD**	79.5 ± 13.9	81.2 ± 12.7	84.2 ± 15.3	89.8 ± 15.7	0.001
**Waist to hip, Mean ± SD**	0.8 ± 0.8	1.4 ± 0.7	1.3 ± 0.7	1.1 ± 0.6	0.011
**FBS, mg/dL, Mean ± SD**	106 ± 34	103 ± 31	112 ± 38	113 ± 40	0.061
**Serum insulin, mg/dL, Mean ± SD**	10.8 ± 7.4	11.4 ± 6.5	13 ± 6.5	15.2 ± 7.2	0.004
**HbA1c, Mean ± SD**	5.9 ± 1	5.9 ± 1.1	6.3 ± 1.3	7.8 ± 10.3	0.046
**Cholesterol, mg/dL, Mean ± SD**	211 ± 64	201 ± 43	189 ± 41	209 ± 54	0.011
**Triglyceride, mg/dL, Mean ± SD**	246 ± 113	193 ± 115	210 ± 139	242 ± 151	0.061
**HDL, mg/dL, Mean ± SD**	42.5 ± 12.4	46.7 ± 10.4	45.5 ± 10.7	45.1 ± 10.7	0.432
**LDL, mg/dL, Mean ± SD**	119 ± 43	118 ± 37	104 ± 33	120 ± 43	0.004

Abbreviations: BMI, body mass index; FBS, fasting blood glucose; HbA1c, hemoglobin A1c; HDL, high density lipoprotein; HOMA, homeostasis model assessment ; LDL, low density lipoprotein; NAFLD, non-alcoholic fatty liver disease.

**Table 2:** Univariate Linear Regression Analysis of Study Variables With Degree of NAFLD Ultrasonography-Based Grading

	Beta	r	95% Confidence Interval		P value
			Lower Bound	Upper Bound	
**Weight, Kg**	0.201	<0.011	0.005	0.016	< 0.001
**Abdominal girth, cm**	0.136	0.009	0.002	0.016	0.007
**Pelvic girth, cm**	0.109	0.008	0.001	0.014	0.032
**FBS, mg/dL**	0.119	0.003	< 0.001	0.005	0.018
**Serum insulin, mg/dL**	0.199	0.023	0.011	0.035	< 0.001
**HbA1C**	0.134	0.025	0.005	0.044	0.012
**Cholesterol, mg/dL**	-0.011	< 0.001	-0.002	0.001	0.827
**HDL, mg/dL**	-0.036	-0.003	-0.010	0.005	0.475
**LDL, mg/dL**	-0.59	-0.001	-0.003	<0.001	0.25
**Triglyceride, mg/dL**	0.099	0.001	<0.001	<0.001	0.049
**AST, mg/dL**	0.123	0.005	<0.001	0.009	0.015
**ALT, mg/dL**	0.113	0.003	0.000	0.005	0.026
**HOMA**	0.169	0.266	0.1	0.432	0.002
**Systolic blood pressure, mmHg**	0.047	0.002	-0.002	0.007	0.357
**Diastolic blood pressure, mmHg**	0.058	0.005	-0.004	0.015	0.25

Abbreviations: ALT, alanin aminotransferase; AST, aspartate aminotransferase; FBS, fasting blood glucose; HbA1c, hemoglobin A1c; HDL, high density lipoprotein; HOMA, homeostasis model assessment; LDL, low density lipoprotein.

## 5. Discussion

In this study, we found that higher; HbA1C, hypertriglyceridemia and body weight levels, are independently associated with higher ultrasonographic NAFLD grades. On the other hand, no association was found between; serum insulin levels, incidental fasting blood glucose, BMI, systolic or diastolic blood pressure, total cholesterol, HDL, or LDL cholesterol levels. An interesting finding was that in a univariate analyses; FBS, serum insulin, liver enzymes, and HOMA, had a significant association with NAFLD scores, but important variables such as; cholesterol values and blood pressure, had no significant effect on NAFLD grading. The pathogenesis of NAFLD has not been precisely defined, although strong evidence has been proposed for some contributing factors (5-8). In one study, Vehmas et al. (8) reported that both systolic and diastolic blood pressure could predict liver echogenicity in NAFLD patients. In contrast, we found no effect for blood pressure on sonographic grading of NAFLD severity; on the other hand, the findings of the two studies similarly reported a significant effect for hypertriglyceridemia and no effect for hypercholesterolemia.

In our study, a multivariate analysis showed that HbA1c and not serum glucose was independently associated with liver echogenicity. A poor correlation between serum FBS levels and liver echogenicity in NAFLD patients has been reported previously (8). This result probably reflects the finding that only long term control of serum glucose levels, which is usually measured by HbA1C, can prevent NAFLD related liver damage. Although this is the first study reporting this finding, higher HbA1C levels in diabetic patients have previously been associated with NAFLD development (7). Another study has also shown that high HbA1C levels in non-diabetic individuals are associated with NAFLD development (5). In addition, insulin resistance has also been associated with NAFLD disease (9). In the current study, a multivariable analysis showed no independent effect for HOMA with NAFLD grades. One explanation for this observation lies in the multifactorial nature of NAFLD pathogenesis. It has been demonstrated that an increase in plasma lipid levels leads to a decrease in hepatic insulin clearance, which results in insulin resistance and hyperinsulinemia (6).

In conclusion, diabetic patients might be able to reduce liver damage from NAFLD by controlling their HbA1C through the lower ranges. Hypertriglyceridemia and body weight are the other implicating factors which exacerbate hepatic echogenicity in the NAFLD patient population. We recommend future prospective studies and clinical trials to confirm our findings.
